# Lung Tissue Microbiome in NSCLC Patients: Metabarcoding Analysis Identifies *Escherichia-Shigella* as an Abundant Taxon

**DOI:** 10.3390/cancers18132105

**Published:** 2026-06-29

**Authors:** Piotr Machnicki, Karolina Czarnecka-Chrebelska, Jacek Kordiak, Krzysztof Lewandowski, Filip Bielec, Tomasz Płoszaj, Ewa Brzeziańska-Lasota, Dorota Pastuszak-Lewandoska

**Affiliations:** 1 Department of Microbiology and Laboratory Medical Immunology, Medical University of Lodz, 90-151 Lodz, Poland; piotr.machnicki@umed.lodz.pl (P.M.); filip.bielec@umed.lodz.pl (F.B.); 2 Department of Biomedicine and Genetics, Medical University of Lodz, Mazowiecka 5, 92-215 Lodz, Poland; karolina.czarnecka@umed.lodz.pl (K.C.-C.); ewa.brzezianska@umed.lodz.pl (E.B.-L.); 3 Department of Thoracic, General and Oncological Surgery, Medical University of Lodz, 90-151 Lodz, Poland; jacek.kordiak@umed.lodz.pl (J.K.); krzysztof.jerzy.lewandowski@umed.lodz.pl (K.L.); 4 Department of Clinical Genetics, Medical University of Lodz, 90-151 Lodz, Poland; tomasz.ploszaj@umed.lodz.pl

**Keywords:** lung cancer, non-small cell lung cancer, NSCLC, microbiome, *Escherichia-Shigella*, oncobiome

## Abstract

**Background**: Non-small cell lung cancer (NSCLC) remains the leading cause of cancer-related mortality worldwide despite advances in diagnosis and treatment. Increasing evidence suggests that alterations in the lung microbiome may contribute to NSCLC development and progression; however, findings remain inconsistent due to heterogeneous biological materials and methodological differences among studies. Therefore, this study aimed to characterize the lung tissue microbiome in NSCLC using a paired tissue-based approach. **Methods**: Thirty-two patients with NSCLC were enrolled. For each patient, two samples were collected: primary tumor tissue and matched macroscopically unchanged adjacent lung tissue. The V3-V4 region of the 16S rRNA gene was amplified and sequenced, followed by bioinformatic analysis using the QIIME2 pipeline. **Results**: Tumor tissues demonstrated lower alpha (Shannon H = 9.60, q = 0.001) and beta (Jaccard pseudo-F = 1.26, q = 0.015) diversity compared with adjacent controls, indicating reduced microbial complexity within the tumor microenvironment. *Escherichia-Shigella* was the most abundant detected genus (~12%) in both groups, although without a statistically significant difference. Analysis of microbiome variation in relation to spatial distance between sampled tissues revealed a strong trend toward significance (*p* = 0.07) with a substantial effect size (R^2^ = 0.207). **Conclusions**: The observed microbiome alterations in NSCLC were more evident at the ecological level than in overall taxonomic composition, supporting a model of microbial community simplification rather than complete compositional replacement. Our findings also suggest that tumor-adjacent lung tissue may not represent a fully neutral control due to the local field effect. The relatively high abundance of *Escherichia-Shigella* indicates that this taxon may warrant further investigation in NSCLC microbiome studies.

## Simple Summary

A growing body of research suggests that microorganisms living in the human body may influence the development and progression of lung cancer, but existing findings are inconsistent due to differences in research designs. In this study, we aimed to address this issue by analyzing tumor tissue and adjacent lung tissue from the same patients, allowing for more accurate comparisons. Using advanced DNA sequencing technology, we investigated which bacteria are present in the lung tumor environment and how they interact. Our results showed that the microbial community is less diverse in cancerous tissue, and this simplification extends beyond the tumor itself. We also identified a potentially harmful, dominant genus *Escherichia-Shigella*, in both tumor and surrounding tissue, suggesting that it may serve as a useful marker for future research. These findings may help improve the understanding of the role of microorganisms in lung cancer and support the development of new diagnostic approaches.

## 1. Introduction

Lung cancer is the most common type of cancer with the highest mortality rate worldwide and represents a major global health challenge. According to the World Health Organization (WHO) estimates from 2022, nearly 2.5 million new cases were diagnosed globally, including over 30 thousand cases in Poland alone [1].

Lung cancers are broadly classified into two major histological groups: small cell lung cancer (SCLC), accounting for approximately 15% of cases, and non-small cell lung cancer (NSCLC), which constitutes about 85% of diagnoses. NSCLC is further subdivided by the WHO into three major subtypes: adenocarcinoma, squamous cell carcinoma, and large cell carcinoma. SCLC is characterized by rapid growth, early metastasis, and limited surgical eligibility, whereas NSCLC generally exhibits slower progression and broader therapeutic options [2,3]. Because NSCLC, unlike SCLC, is more often diagnosed at stages enabling surgical resection, this allows for paired sampling of tumor tissue and adjacent non-malignant lung tissue, which is essential for controlled microbiome comparisons. Additionally, the histological heterogeneity of NSCLC offers an additional analytical dimension, enabling subtype-specific microbial analyses that are not as readily feasible in SCLC.

Despite substantial advances in molecular diagnostics, targeted therapies, and immunotherapy, NSCLC is characterized by particularly poor survival rates due to late-stage diagnosis in many patients, because there are almost no noticeable symptoms in the early stages. Prevention strategies mainly include smoking cessation, reduction of environmental exposures, and low-dose computed tomography (LDCT) screening in high-risk populations. Current therapeutic approaches depend on tumor subtype and stage and include surgery, chemotherapy, radiotherapy, targeted therapies, and immunotherapy [2,4].

Numerous tissue- and blood-based biomarkers are currently used in lung cancer diagnosis and treatment stratification. Molecular profiling of tumor tissue includes EGFR, ALK, ROS1, KRAS, and PD-L1 assessment, while liquid biopsy approaches based on circulating tumor DNA (ctDNA), circulating tumor cells, and extracellular vesicles are increasingly explored as minimally invasive diagnostic and predictive tools. However, the identification of novel biomarkers and mechanisms involved in NSCLC pathogenesis is still of great clinical importance [2,4].

Cigarette smoking, chronic lung diseases and air pollution are the main well-known risk factors for lung cancer [5]. Nevertheless, increasing attention has been directed toward the potential role of the lung microbiome in the development and progression of this disease. Dysbiosis has increasingly been implicated in the development and progression of multiple malignancies, including colorectal, pancreatic, breast, and lung cancer. The most extensively studied association between the microbiome and cancer involves the gut microbiota and its contribution to colorectal carcinogenesis, where sustained dysbiosis and close interactions with the host immune system promote chronic inflammation and tumor initiation [6].

An analogous hypothesis was proposed for lung cancer; however, it was not until the first decade of the 2000s, with the development of next-generation sequencing technologies, that it became possible to begin exploring these theories in depth. The overturning of the long-held paradigm of lung sterility has led to the recognition of a complex and dynamic pulmonary microenvironment. The most frequently reported phyla of a healthy lung microbiome are Bacteroidota and Bacillota, followed by Pseudomonadota, Actinomycetota and Fusobacteriota, while on the genus level, the most prevalent are *Prevotella*, *Streptococcus*, *Veilonella*, *Acinetobacter* and *Corynebacterium* [7,8,9,10,11].

Subsequent investigations across a range of pulmonary diseases have demonstrated substantial alterations in microbial community structure, often characterized by reduced richness and diversity. Importantly, each disease entity appears to exhibit a distinct microbial profile. For example, asthma is commonly associated with an overrepresentation of Pseudomonadota, particularly *Haemophilus* spp., and an underrepresentation of Bacteroidota, especially *Prevotella* spp. [8], and lung cancer appears to follow a similarly disease-specific pattern. Analyses of lung tumor microbiome frequently report alterations in alpha diversity indices. Several studies have documented reduced microbial diversity in lung cancer samples [12,13,14,15], whereas others have found no significant differences [16,17,18].

Inconsistent results are also evident in reported taxonomic shifts. At the phylum level, the most commonly described changes involve Pseudomonadota, Bacillota, Bacteroidota, and Actinomycetota [12,13,14,15,16,17,18,19,20]. This variability becomes even more pronounced at the genus level and is further influenced by the sampling method employed. Lee et al., 2016 [19], reported an increased abundance of *Veillonella* and *Megasphaera* in bronchoalveolar lavage fluid (BALF). Similar results were obtained by Zeng et al., 2022 [20], who additionally identified *Streptococcus* to be associated with NSCLC. Seixas et al., 2021 [17], also demonstrated increased frequency of *Streptococcus* and *Prevotella* in lung cancer patients compared with individuals with interstitial lung disease; interestingly, no changes in *Veillonella* abundance were observed. On the other hand, analyses of tumor tissue of smokers and those harboring TP-53 mutations revealed a higher prevalence of *Acidovorax* [16,17,18,19,20,21]. Another frequently reported genus associated with lung cancer is *Acinetobacter*; however, its role remains unclear, as studies have reported both increased and decreased abundance [22].

Although dysbiosis observed within the tumor microenvironment—frequently called the oncobiome—remains incompletely understood, numerous theories have been proposed to explain how microorganisms influence carcinogenesis and cancer progression. One of the leading concepts involves modulation of the host immune system. To maintain lung homeostasis and protect this fragile organ from persistent inflammation, continuous crosstalk occurs between commensal bacteria and immune cells through pattern recognition receptors (PRRs) expressed by alveolar macrophages, dendritic cells, and epithelial cells, which recognize microbial-associated molecular patterns (MAMPs). Under physiological conditions, controlled PRR engagement by commensal microbiota promotes M2 macrophage polarization and supports the expansion of regulatory T cells (Tregs), which together suppress excessive Th17 activity through the production of anti-inflammatory cytokines, such as IL-10 and TGF-β [23,24]. However, this immunoregulatory axis appears to be highly context-dependent. In the setting of dysbiosis, particularly with enrichment of oral-associated taxa in the lower airways, persistent or altered PRR stimulation has been associated with enhanced Th17 mobilization and neutrophilic inflammation, which have already been linked to activation of oncogenic signaling pathways, including MAPK/ERK [23,25,26]. Collectively, these findings suggest that microbial-driven immune reprogramming may shift the pulmonary microenvironment from immune tolerance to chronic inflammation and pro-tumorigenic signaling. Elucidating these pathways and identifying specific bacterial taxa responsible for their activation may contribute to the development of novel diagnostic biomarkers and targeted therapeutic strategies.

Many differences in reported bacterial community changes are often ascribed to heterogeneity of sampling methods and group sizes. Therefore, given the significant discrepancies between published studies and the susceptibility of the low-biomass microbiome to contamination, further studies using rigorously controlled methods are necessary. Furthermore, direct comparison of tumor tissue with adjacent, non-malignant lung tissue may provide a more reliable characterization of cancer-related microbial changes. The aim of our study was to contribute to the growing body of evidence on bacterial-lung tumor interactions by comparing the microbial composition of tumor tissue with adjacent macroscopically unchanged tissue, which served as a control representing non-malignant lung tissue, while implementing rigorous anti-contamination measures throughout both laboratory processing and bioinformatic analysis.

## 2. Materials and Methods

### 2.1. Patient Selection and Sample Collection

The research material was collected in the Department of Thoracic, General, and Oncological Surgery at the Medical University of Lodz. The inclusion criteria comprised a diagnosis of NSCLC in a positron emission tomography (PET) or computed tomography scan (CT), qualifying for the surgery. Only patients from whom paired tumor tissue and adjacent non-malignant lung tissue were obtained were eligible for inclusion in the microbiome analyses. Such paired sampling is particularly valuable for controlled microbiome comparisons, as it minimizes inter-individual variability. Thirty-two patients were classified for the study; all of them were diagnosed with NSCLC and underwent tumor resection through lobectomy (*n* = 18), segmentectomy (*n* = 10), pneumonectomy (*n* = 2), or wedge resection (*n* = 2). Clinical and demographic data were collected retrospectively from medical records. Information regarding smoking status, family history of cancer, cancer stage, and histopathological diagnosis was obtained when available. The cohort consisted of 17 males and 15 females aged 47 to 89. Regarding smoking status, 30 patients were current or former smokers, and two were never-smokers. Information on familial cancer burden was available for all patients enrolled: 12 patients reported a positive family history of malignancy, while 20 reported none. Histopathological classification revealed that 15 tumors were adenocarcinomas and 15 were squamous cell carcinomas. One patient was diagnosed with atypical carcinoid, and one case was classified as undifferentiated non-small cell lung cancer. A summary of the clinical and demographic characteristics of the study cohort is presented in Supplementary Materials Table S1. Exclusion criteria included a history of other malignancies, previous chemotherapy or radiotherapy, as well as active infectious disease. Prior to inclusion, all patients provided written informed consent. The study was conducted in accordance with the Declaration of Helsinki and approved by the Bioethics Committee of the Medical University of Lodz (approval No RNN/11/22/KE). During surgery, two tissue samples were collected from each patient; tumor samples were cut from the center of malignant tissue, while adjacent non-malignant lung tissue (serving as control tissue) was obtained as far as possible from macroscopically changed tissue, and this distance depended on the type of procedure applied. Each sample was frozen immediately after surgery at −80 °C.

### 2.2. DNA Extraction and Sequencing

Prior to DNA extraction, 30 mg of each sample was cut into smaller pieces using a sterile scalpel blade. Genetic material was isolated using the Qiagen DNeasy^®^ Blood & Tissue Kit (catalogue no. 69504, Qiagen^®^, Hilden, Germany) according to the manufacturer’s instructions. To increase the yield of extracted DNA from Gram-positive bacteria, samples underwent pre-treatment in Enzymatic-Lysis buffer, as described in the kit’s user manual. A negative control was included in each extraction batch. The concentration and quality of the obtained DNA were evaluated by spectrophotometric measurement followed by quantification with QuantiFluor^®^ dsDNA System (Promega^®^, Madison, WI, USA) to increase accuracy. All samples were classified for Next Generation Sequencing (NGS) using hypervariable V3-V4 regions of the 16S rRNA gene. Specific primer sequences 341F and 785R [27] were used for amplification and library preparation according to the Illumina^®^ manual: 16S Metagenomic Sequencing Library Preparation—Preparing 16S Ribosomal RNA Gene Amplicons for the Illumina MiSeq System. PCR was conducted under conditions specified by the manufacturer (initial denaturation at 95 °C for 3 min followed by 25 cycles of 3: 95 °C for 30 s, 55 °C for 30 s and 72 °C for 30 s, with the final extension at 72 °C for 5 min). Sequencing was performed on the MiSeq platform using paired-end technology (2 × 300 bp) with the Illumina^®^ v3 kit (5200 Illumina Way, San Diego, CA, USA).

### 2.3. Bioinformatics Analysis

**Figure 1:**
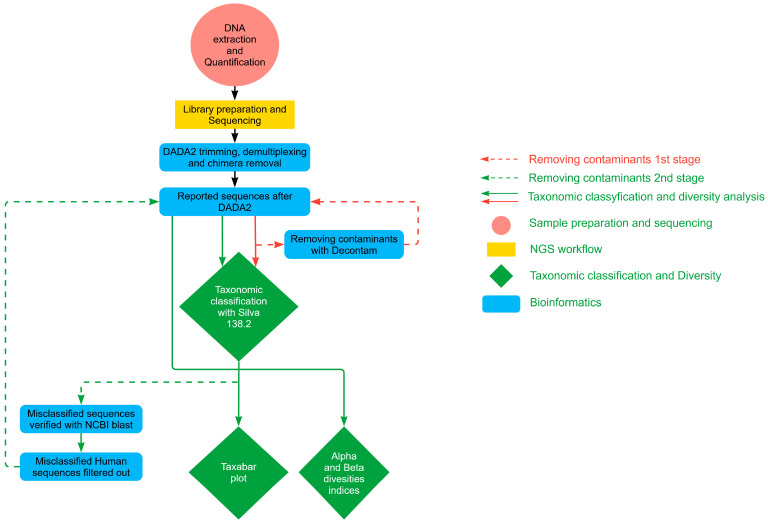
Implemented workflow.

Bioinformatics analysis was performed using the QIIME2 workflow [28,29] with the use of the QIIME 2 DADA2 package version 2025.10.1 [30], which enables the identification of unique Amplicon Sequence Variants (ASVs). ASV analysis was chosen over the OTU (Operational Taxonomic Unit) method to minimize error introduced by clustering sequences into a consensus sequence and to enable easier and more precise results in comparison with upcoming studies. In short, demultiplexed sequences were trimmed, denoised, merged and underwent chimeric sequences removal. Next, the remaining sequences were run through a decontamination tool: Decontam [31]—part of the qiime2 quality control package. Due to a high human-bacteria DNA ratio, the prevalence method was chosen to remove contaminants, and a threshold of 0.5 was set. The obtained ASVs were then compared with the reference Silva database (version 138.2) [32] to assign microbial taxonomy. Taxonomic identification was done with the QIIME2 feature-classifier-sklearn package version 2025.10.1 and, when possible, included classification to the species level. Due to the high human DNA burden of the samples, sequences classified as: “Eukaryota”, “Unassigned” or “d_Bacteria;_” were individually cross-checked with the NCBI blast database, and after identification as human DNA, filtered out from further analysis. Sequence frequencies of each step are presented in Supplementary Materials Table S2. Detailed taxa bar plot charts were generated to visualize the results, presenting the percentage distribution of taxa across the samples. To assess the adequacy of the sampling depth, alpha rarefaction curves were generated, and biodiversity indices were calculated. A schematic representation of the implemented workflow is presented in Figure 1.

Additionally, based on QIIME2 core metrics results, a linear mixed-effects model (LMM) with use of RStudio (v. 2026.01.1+403), lme4 library (v. 1.1-38) was applied to evaluate the potential “field effect” associated with the surgical margin size, considering the type of procedure as reflecting differences in the distance of sampled tissue from the tumor. Tissue type and surgical procedure were treated as fixed effects, while patient ID was included as a random effect to account for inter-individual variability and the paired structure of the data. Moreover, Bray–Curtis distances between tumor and control tissues were calculated in relation to the type of procedure, and visualized using Principal Coordinates Analysis (PCoA).

## 3. Results

Through all samples analyzed after denoising, sequence counts per sample ranged from 11,009 to 142,118 reads, with a median of 38,251 reads. The first step of the decontamination procedure identified 16 contaminant features, 32,905 non-contaminant features, corresponding to a contaminant proportion of 0.05% of all detected features, while 22,292 features were assigned an NA score, with a median of 189 reads per sample. Rarefaction analysis demonstrated a rapid increase in observed features at low sequencing depths, followed by a gradual plateau for the majority of samples (Supplementary Materials Figure S3). Based on these results, a rarefaction depth of 200 sequences per sample was selected for downstream analyses.

**Figure 2:**
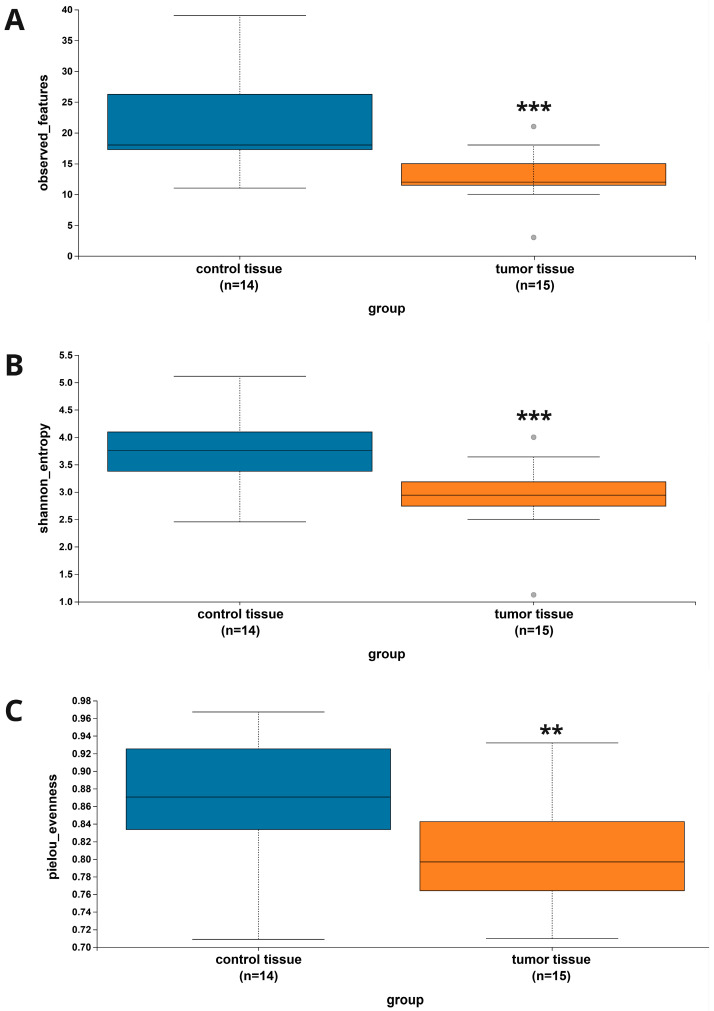
Comparison of alpha diversity between tumor and control tissue samples, using the Kruskal–Wallis (pairwise) test. Control tissue *n* = 14, tumor *n* = 15. ** *p* < 0.01, *** *p* < 0.001 (**A**) observed features (H = 10.441, *p* = 0.001, q = 0.001), (**B**) Shannon diversity index (H = 9.601, *p* = 0.001, q = 0.001), and (**C**) evenness (H = 4.573, *p* = 0.03, q = 0.03). Dots beyond the whiskers represent outliers.

Alpha diversity differed significantly between control tissue and tumor samples (see Figure 2). Tumor samples exhibited a significantly lower number of observed features compared to control tissue (H = 10.44, q = 0.001), indicating reduced taxonomic richness. Similarly, Shannon diversity was significantly decreased in tumor samples (H = 9.60, q = 0.001), reflecting differences in both richness and relative abundance distribution. Evenness was also significantly reduced in tumor samples compared to control tissue (H = 4.57, q = 0.032).

**Figure 3:**
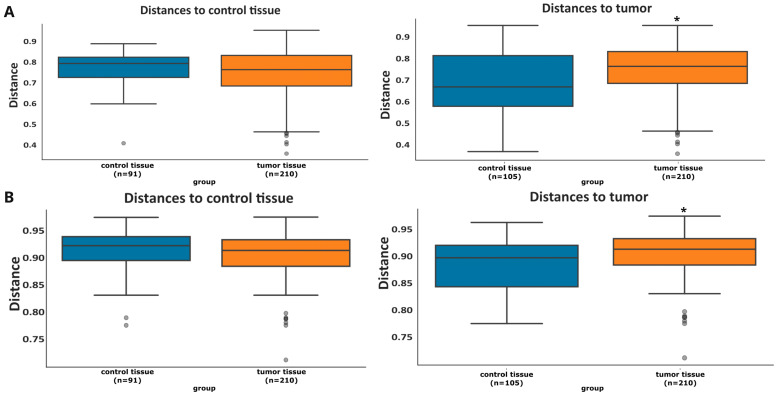
Comparison of beta diversity between tumor and control tissue samples: (**A**) Jaccard index (PERMANOVA: pseudo-F = 1.26, *p* = 0.015, q = 0.015); (**B**) Bray–Curtis dissimilarity index (PERMANOVA: pseudo-F = 1.65, *p* = 0.03, q = 0.03). * *p* < 0.05. Dots beyond the whiskers represent outliers.

Beta diversity analyses further revealed significant differences in microbial community composition between control tissue and tumor samples, depending on the distance metric applied (see Figure 3).

When comparing presence-absence of taxa, the Jaccard index showed a significant separation between the control and tumor microbiomes (PERMANOVA: pseudo-F = 1.26, *p* = 0.015, q = 0.015). These results were supported by the Bray–Curtis dissimilarity index, which also indicated a significant difference between the two groups (PERMANOVA: pseudo-F = 1.65, *p* = 0.025, q = 0.025). In contrast, phylogeny-based distance metrics, including unweighted and weighted UniFrac, did not reveal statistically significant differences between macroscopically unchanged and tumor samples, with the ordination plots demonstrating substantial overlap and dispersion of samples between groups (Supplementary Figure S4).

While comparing alpha and beta diversity of samples in relationship with age, gender, smoking habits (pack-years vs. non-smokers), presence or absence of cancer history in family, cancer type (squamous cell carcinoma or adenocarcinoma) and tumor stage based on the VIII edition of TNM classification, no significant statistical differences have been found.

**Figure 4:**
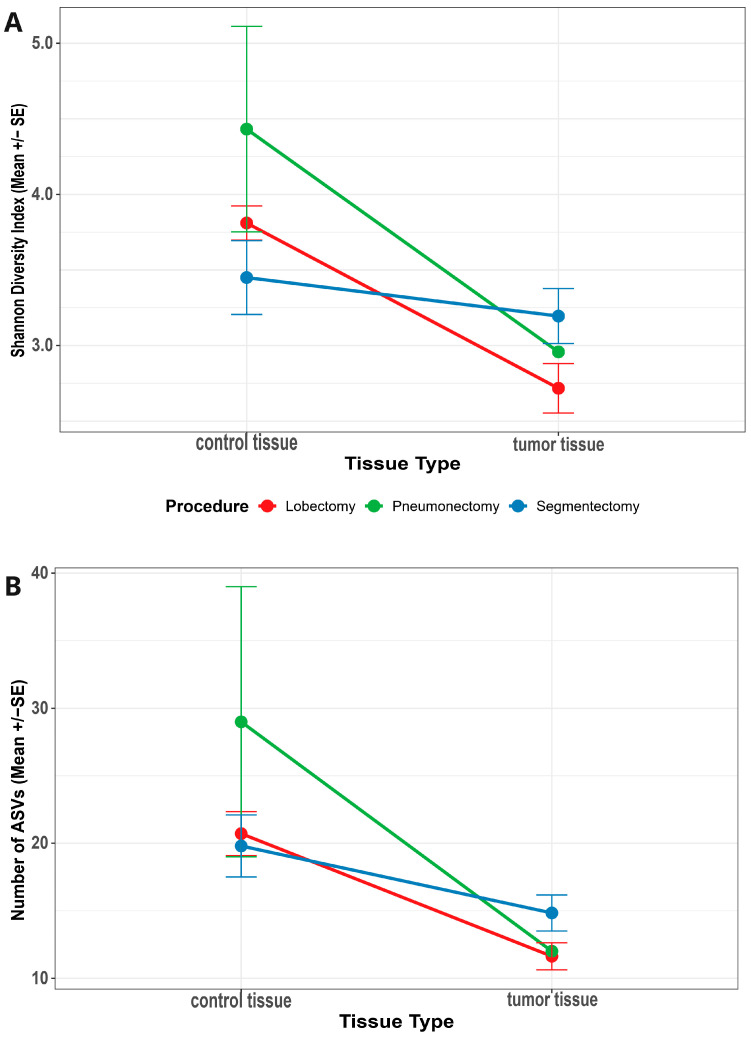
Interaction effect type of procedure vs. tissue microbiome. (**A**) Shannon Diversity; (**B**) Observed features. Points represent mean values, and error bars represent ± SD. The Y-axis shows the index values, and the X-axis shows the tissue types.

LMM analysis of the impact of surgical margin on alpha diversity showed a clear interaction between the type of surgical procedure and the degree of diversity loss, although it did not reach statistical significance (see Figure 4).

**Figure 5:**
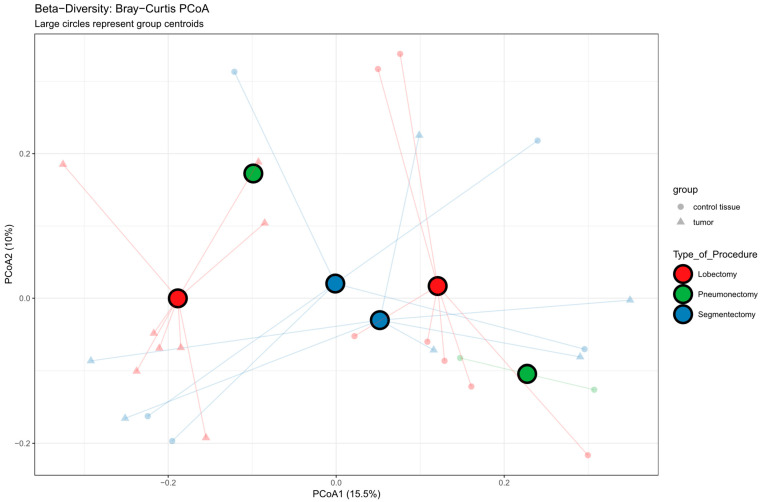
Microbiome diversity based on Bray–Curtis PCoA in a relationship with the type of tissue and type of procedure. Large points indicate group centroids, and lines connect individual samples to their respective group centroids.

Bray–Curtis Beta diversity analysis with relationship to type of procedure implemented, showed a strong trend towards statistical significance (*p* = 0.07; R^2^ = 0.207), as presented in Figure 5.

**Figure 6:**
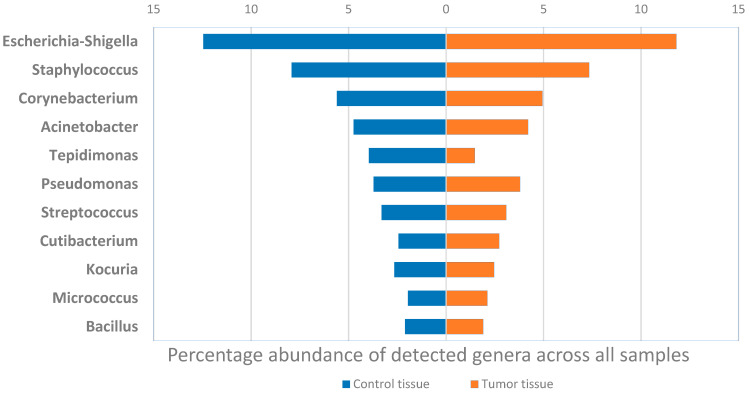
Most frequently observed genera. No statistical significance between the studied groups was found.

Detailed taxonomic composition, divided into tumor and control tissue with resolution up to phylum and genus level, is presented in Supplementary Materials Figures S5.1–S5.4. The most abundant phyla across all samples were Pseudomonadota, Bacillota, Actinomycetota and Bacteroidota, regardless of the sample source. The most abundant genera identified across the analyzed sample set, both in tumor and adjacent macroscopically unchanged lung tissues, are summarized in Figure 6.

## 4. Discussion

The aim of the study was to analyze the microbiome profile of lung tumor tissue and compare it with that of control tissue in patients with non-small cell lung cancer (NSCLC) using next-generation sequencing (NGS). Particular attention was paid to contamination control procedures, as well as to minimizing sample source heterogeneity—both of which are common limitations in similar studies. In many previous reports, comparisons were performed between heterogeneous sample types, e.g., BALF vs. tumor tissue, used in different studies, or were based on relatively small cohorts. To address these limitations, our study was designed to include two types of tissue samples obtained from each NSCLC patient, i.e., primary tumor tissue and the corresponding macroscopically unchanged adjacent tissue, used as a control, thereby ensuring a balanced comparison. Rigorous contamination surveillance was implemented throughout both the laboratory workflow and the bioinformatic analysis to enhance the reliability and robustness of the results.

In a healthy lung tissue, the average number of bacteria has been estimated at 10^3^ to 10^5^ per 1 g of tissue [33], which entitles it to be defined as a low-biomass material. Additionally, as found by Nejman et al., 2020 [34], who analyzed over 1500 different tumors and adjacent control tissues, intratumor bacteria are localized within immune or tumor cells. Our concern was the high ratio of human DNA to bacterial DNA in the studied tissue samples. Therefore, the implementation of rigorous anti-contaminant policies and procedures—aimed at retaining only truly bacterial-derived sequences—led to a drop in reads from tens of thousands to hundreds. However, such outcomes are commonly observed when analyzing low microbial load samples [16,24,35] such as tissue, and our results should be interpreted in the context of low-biomass microbiome profiling.

In fact, the applied alpha rarefaction analysis showed that increasing sequencing depth beyond 200 reads per sample did not result in significant differences, yielding only marginal gains in observed diversity. This supports the suitability of the chosen normalization threshold for comparative analyses. The obtained alpha diversity indices indicate an increased dysbiosis in lung cancer tissue, characterized by reduced richness and evenness. These findings are consistent with the typically reported lung cancer microbiome composition [12,14].

We did not observe significant associations between microbiome diversity and age, sex, smoking status, family history, histological subtype, or tumor stage. In this respect, our findings are consistent with those of previous studies by Jin et al., 2019 [13], Chen et al., 2022 [14], Dong et al., 2022 [16], and Greathouse et al., 2018 [21], who also reported no overall changes in microbial diversity in relation to environmental, demographic, or histopathological factors. Although these negative findings may partly reflect limited power for subgroup analyses, they may also suggest that the principal microbiome signal detected in our study is more closely related to the tumor-associated environment itself rather than to demographic and clinical variables. However, there are reports describing differential abundance of specific taxa, for example, enrichment of *Acidovorax* in smokers with lung cancer compared to non-smoking patients [16,21].

Interestingly, despite detected differences, the microbiomes of macroscopically unchanged and tumor tissues turned out to be phylogenetically similar, and in this respect, our results are in accordance with those obtained by Dong et al., 2022 [16], Seixas et al., 2021 [17] and Zhuo et al., 2020 [18], who also did not identify significant alpha diversity differences between control lung tissue and tumor tissue. An additional point emerging from our results is that the observed tumor-associated dysbiosis appears to be driven more by ecological simplification rather than by a profound replacement of the phylogenetic core microbiome. Tumor samples were characterized by a generally reduced microbial diversity, reflected both in a lower number of detected taxa and the less even distribution of their relative abundance. While differences between tumor and corresponding macroscopically unchanged adjacent tissue were evident when considering compositional dissimilarity, they were not apparent when accounting for phylogenetic relationships among taxa.

The most common phyla identified in our study, i.e., Pseudomonadota, Bacillota, Actinomycetota, and Bacteroidota, are consistent with previous reports [24,36]. However, when comparing their ranking of the identified phyla with findings from other studies (Lee et al., 2016 [19], Liu et al., 2018 [12] or Zeng et al., 2022 [20]), it becomes evident that their relative abundances vary. Along with biodiversity data, this pattern indicates selective enrichment or loss of specific taxa within a generally similar phylogenetic community. Whether these changes influence lung tumor development and progression remains uncertain.

Moreover, a similar pattern can be observed at the genus level, where the most frequently reported genera, i.e., *Staphylococcus*, *Corynebacterium*, *Acinetobacter*, *Pseudomonas*, and *Streptococcus*, were also among the most commonly identified in our study. Interestingly, approximately 12% of identified bacteria were classified as *Escherichia*-*Shigella*, the potentially pathogenic Gram-negative bacteria. To the best of our knowledge, these bacteria were first detected specifically in lung tumor tissue by Dumont-Leblond et al., 2021 [37]; however, they did not find them in adjacent macroscopically unchanged tissue. In the same year, the presence of *Escherichia-Shigella* was also confirmed in BALF of patients with diagnosed lung cancer [17].

Only a recent study by Sha et al., 2025 [38] reported an increased abundance of *Escherichia*-*Shigella* both in tumor tissue and, although in lower quantities, in adjacent non-malignant tissue in NSCLC patients. Moreover, the abundance of these bacteria positively correlated with tumor size and the expression of Ki67, a marker of cell proliferation. The potential involvement of the microbiome in lung cancer carcinogenesis is currently explained mainly by chronic inflammatory stimulation, disruption of the local immune response, and the direct impact of bacterial metabolites and toxins on cancer cells. Virulence factors produced by pathogenic bacteria may penetrate the mucus barrier and trigger an inflammatory reaction [39]. In the case of the *Escherichia-Shigella* genus, lipopolysaccharide (LPS), a Gram-negative bacterial endotoxin, appears to play a particularly important role. Indeed, in vivo and in vitro analyses performed by Sha et al., 2025 [38] demonstrated that the increased abundance of *Escherichia*-*Shigella*, together with other *unclassified_Enterobacteriaceae*, resulted in elevated levels of intratumor LPS, thereby promoting tumor proliferation through chronic activation of the inflammatory TLR4-mTOR-NF-ĸB-IL-6 axis. In accordance with these findings, we also detected *Escherichia*-*Shigella* in both tumor and control tissue, although without statistically significant differences.

With the development of high-throughput sequencing technology, the functional roles of the intratumoral microbiota in tumor development are gradually being elucidated. However, as already mentioned, the specific communities associated with lung carcinogenesis, as well as the underlying mechanisms, remain mostly unclear. Only a few studies have proposed mechanistic explanations for the role of individual taxa. For example, upregulation of PI3K and ERK signaling pathways has been observed in association with *Streptococcus*, *Veillonella*, and *Prevotella* [17,40]. In turn, *Acidovorax* has been suggested to promote tumorigenesis through mutations in the tumor suppressor gene TP53 [21]. However, for other genera frequently detected in lung cancer samples, no clear mechanism has yet been proposed [40].

As mentioned above, the presence of *Escherichia*-*Shigella* was revealed in both types of tissues analyzed. The same applies to other bacteria identified both in the primary tumor and in adjacent tissue. The concept that molecular and genetic changes extend beyond the visible tumor and can be detected already in tissues that appear histologically unchanged is called the “field effect” or “field cancerization” [41,42,43]. Our findings, both LMM results and taxonomic identification in subjected groups, appear to support that phenomenon and even extend it beyond genetic alterations to the tumor-associated microbiome that outspreads macroscopically altered tissue. Furthermore, we obtained interesting results by estimating the relative contribution of individual taxa depending on the distance between the tumor and the control tissue. The analysis, although indirectly—as it was based on the type of procedure—showed a strong tendency towards statistical significance and a substantial effect size, indicating that over 20% of the variability in microbial composition could be explained by the factor studied. Our findings suggest that the interpretation of microbiome in tumor-adjacent tissue may depend not only on histopathological assessment, but also on sampling geometry and surgical context. Together with the observed presence of *Escherichia*-*Shigella* across both tissue types, it raises the possibility of their association with ongoing carcinogenic processes and suggests that *Escherichia*-*Shigella* may represent a potential lung tumor biomarker. However, further studies are required to confirm this relationship.

This study has several notable strengths and novel aspects. Importantly, microbiome testing is performed directly on lung tissue and paired adjacent non-tumor tissue collected from the site of carcinogenesis itself, thereby minimizing the risk of cross-contamination from other respiratory sources. While previous studies investigating the lung microbiome have frequently relied on samples such as sputum or bronchoalveolar lavage fluid (BALF), relatively few studies have analyzed resected lung tissue directly, which may more accurately reflect the local tumor microenvironment and microbial alterations associated with lung carcinogenesis. The use of paired tumor and adjacent tissue samples from the same patient enabled intra-individual comparisons, thereby reducing inter-patient variability and strengthening the internal validity of the findings. Furthermore, the relatively large cohort size enhanced the robustness and generalizability of the results. Importantly, strict anti-contamination procedures were implemented throughout sample processing and sequencing workflows, ensuring that only high-confidence bacterial sequences were genuinely present in the analyzed tissues.

Nevertheless, several limitations should be acknowledged. The combination of stringent decontamination procedures and the high proportion of host to bacterial DNA required intensive sequence filtering, which resulted in a reduced number of reads per sample and a relatively shallow sampling depth, although it was still sufficient for the planned analysis. Additionally, the study would have benefited from the inclusion of lung tissue from truly healthy, non-cancer controls; however, due to ethical and logistical constraints, such material could not be incorporated into the study design. Future studies should therefore focus on larger multicenter cohorts, the inclusion of appropriate healthy control samples where feasible, deeper sequencing strategies, and functional analyses to further elucidate the potential mechanistic role of the lung microbiome in lung cancer development and progression.

## 5. Conclusions

In this paired tissue-based study, NSCLC tumors showed reduced microbial richness, Shannon diversity, and evenness compared with adjacent macroscopically unchanged lung tissue, indicating decreased microbial complexity within the tumor microenvironment. These differences were more evident in ecological diversity indices than in taxonomic composition, suggesting that NSCLC-associated microbiome alterations may reflect changes in microbial community structure rather than complete replacement of dominant taxa. Our findings also indicate that tumor-adjacent tissue may not represent a fully neutral comparator, as its microbiome composition may be influenced by local tumor-associated effects, sampling distance, and surgical context. Although no statistically significant differences in *Escherichia-Shigella* abundance were observed between tumor and adjacent non-malignant tissues, this genus represented the most abundant detected taxon in both groups. Its relatively high abundance in tumor samples and its previous identification in lung cancer-associated specimens suggest that this taxon may warrant further investigation in NSCLC microbiome studies on potential biomarkers in tumor formation and/or progression.

Further studies involving larger patient cohorts, standardized sampling strategies, and functional microbiome analyses are needed to clarify the biological significance of lung microbiome alterations in NSCLC development and progression.

## Data Availability

The data presented in this study are available on request from the corresponding author. The metabarcoding datasets generated in this study are derived from human lung samples and are therefore subject to ethical and privacy restrictions. Notably, a substantial proportion of the raw sequencing reads are of human origin; thus, the raw metabarcoding data contain not only microbial sequences but also host-derived genetic material, which may carry a risk of participant identification.

## Supplementary Materials

The following supporting information can be downloaded at: https://www.mdpi.com/article/10.3390/cancers18132105/s1, Table S1: Summary of the clinical and demographic characteristics of the study cohort; Table S2: Sequence frequencies of each filtration step; Figure S3: Rarefaction curves; Figure S4: Emperor 3D visualization; Figure S5.1: Taxonomic composition of samples at phylum level -bars; Figure S5.2: Taxonomic composition of samples at phylum level–legend; Figure S5.3: Taxonomic composition of samples at genus level -bars; Figure S5.4: Taxonomic composition of samples at genus level–legend.

## Abbreviations

The following abbreviations are used in this manuscript:

SCLC	Small cell lung cancer
NSCLC	Non-small cell lung cancer
BALF	Bronchoalveolar lavage fluid
PRRs	Pattern recognition receptors
MAMPs	Microbial-associated molecular patterns
PET	Positron emission tomography
CT	Computed tomography scan
NGS	Next Generation Sequencing
QIIME2	Quantitative Insights Into Microbial Ecology 2
ASVs	Amplicon Sequence Variants
OTUs	Operational Taxonomic Unit
LMM	linear mixed-effects model
PCoA	Principal Coordinates Analysis
