# Supplementary material for: Lung Tissue Microbiome in NSCLC Patients: Metabarcoding Analysis Identifies Escherichia-Shigella as an Abundant Taxon
Source: Cancers (Basel). 2026 Jun 29;18(13):2105. doi: 10.3390/cancers18132105 (PMC13359530; doi:10.3390/cancers18132105)
Supplement: Supplementary file 1 [file cancers-18-02105-s001.zip › Supplementary materials Figure S4 Emperor 3D visualization.pdf]

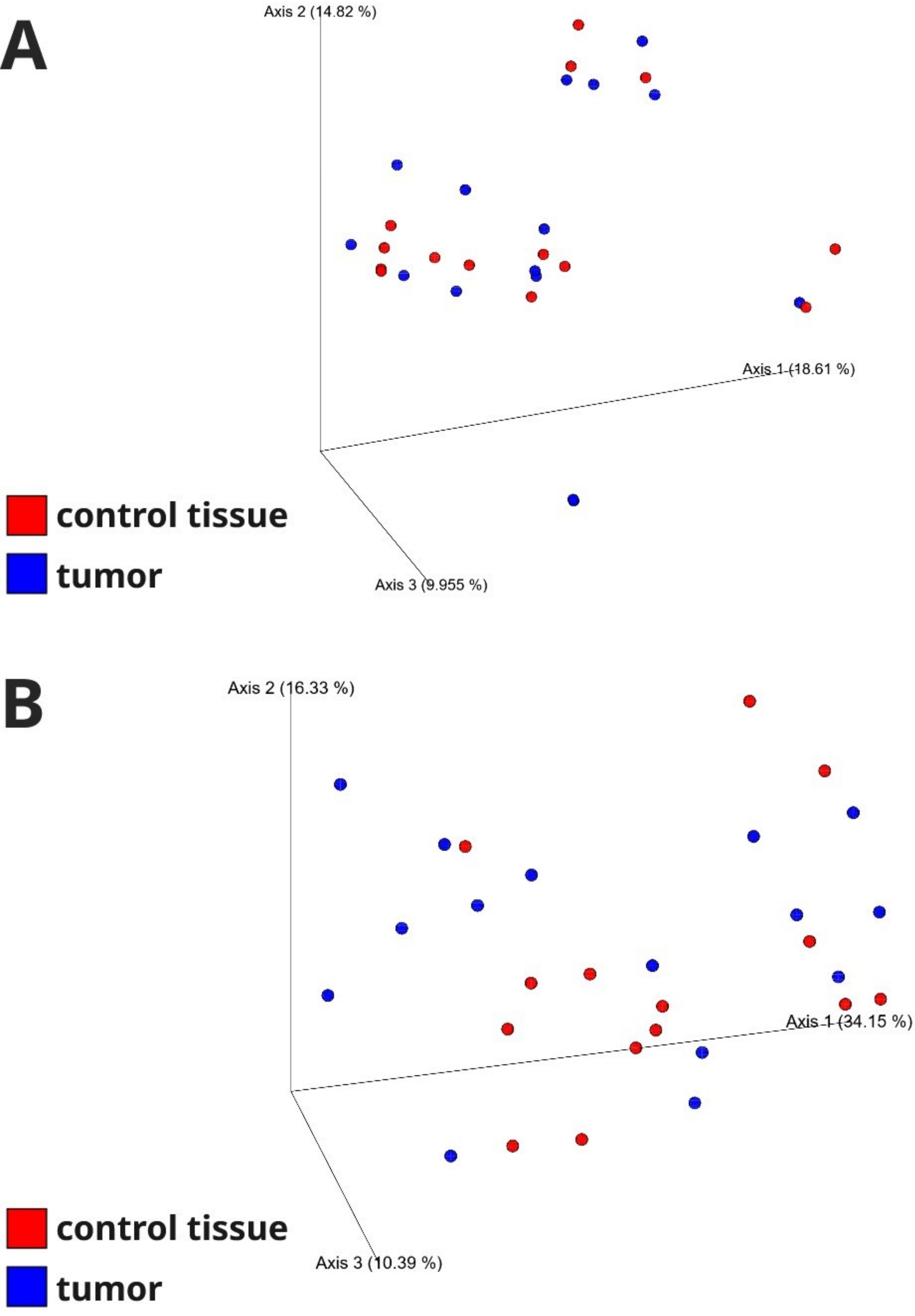

Figure S4. UniFrac-based beta diversity analysis (A: unweighted and B: weighted) of macroscopically unchanged and tumor lung tissue samples, visualized using QIIME2 Emperor plots. No significant differences were observed between groups
