# Supplementary material for: Lung Tissue Microbiome in NSCLC Patients: Metabarcoding Analysis Identifies Escherichia-Shigella as an Abundant Taxon
Source: Cancers (Basel). 2026 Jun 29;18(13):2105. doi: 10.3390/cancers18132105 (PMC13359530; doi:10.3390/cancers18132105)
Supplement: Supplementary file 1 [file cancers-18-02105-s001.zip › Supplementary Materials Table S1 Summary of the clinical and demographic characteristics of the study cohort.pdf]

**CHARACTERISTIC**	**TOTAL *n*=32**
**AGE (YEARS)**	Me=70.3 ± 6.6
**GENDER, *n*(%)**	
- FEMALE	46.88%
- MALE	53.12%
**SMOKING STATUS**	
-NEVER SMOKERS	6%
-SMOKRES	94%
-PACK YEARS	Me=54.4 ± 10.6
**FAMILY HISTORY OF CANCER**	
- YES	37.5%
- NO	62.5%
**CANCER STAGE (VIII)**	
-IA2	15.63%
-IA3	18.75%
-IB	18.75%
-IIA	9.38%
-IIB	28.13%
-IIIA	3.13%
-IIIC	3.13%
-IVA	3.13%
**CANCER TYPE**	
-ADENOCARCINOMA	46.88%
-SQUAMOUS CELL CARCINOMA	46.88%
-OTHER NSCLC	6.25%
**TYPE OF PROCEDURE**	
-LOBECTOMY	56.25%
-SEGMENTECTOMY	31.25%
-PNEUMONECTOMY	6.25%
-WEDGE RESECTION	6.25%

Table S1 Summary of the clinical and demographic characteristics of the study cohort. Categorical variables are presented as percentages, while continuous variables are reported as mean ± standard deviation.
